# Avoiding Systematic Errors in Isometric Squat-Related Studies without Pre-Familiarization by Using Sufficient Numbers of Trials

**DOI:** 10.2478/hukin-2014-0074

**Published:** 2014-10-10

**Authors:** Ekim Pekünlü, İlbilge Özsu

**Affiliations:** 1 Coaching Education Department, Division of Movement and Training Sciences, School of Physical Education and Sports, Ege University, Bornova, İzmir, TÜRKİYE.; 2 Division of Movement and Training Sciences, Institute of Health Sciences, Ege University, Bornova, İzmir, TÜRKİYE.

**Keywords:** isometric testing standards, learning effect, number of trials, maximal isometric strength, testing study assumptions

## Abstract

There is no scientific evidence in the literature indicating that maximal isometric strength measures can be assessed within 3 trials. We questioned whether the results of isometric squat-related studies in which maximal isometric squat strength (MISS) testing was performed using limited numbers of trials without pre-familiarization might have included systematic errors, especially those resulting from acute learning effects. Forty resistance-trained male participants performed 8 isometric squat trials without pre-familiarization. The highest measures in the first “n” trials (3 ≤ n ≤ 8) of these 8 squats were regarded as MISS obtained using 6 different MISS test methods featuring different numbers of trials (The Best of n Trials Method [BnT]). When B3T and B8T were paired with other methods, high reliability was found between the paired methods in terms of intraclass correlation coefficients (0.93–0.98) and coefficients of variation (3.4–7.0%). The Wilcoxon’s signed rank test indicated that MISS obtained using B3T and B8T were lower (p < 0.001) and higher (p < 0.001), respectively, than those obtained using other methods. The Bland-Altman method revealed a lack of agreement between any of the paired methods. Simulation studies illustrated that increasing the number of trials to 9–10 using a relatively large sample size (i.e., ≥ 24) could be an effective means of obtaining the actual MISS values of the participants. The common use of a limited number of trials in MISS tests without pre-familiarization appears to have no solid scientific base. Our findings suggest that the number of trials should be increased in commonly used MISS tests to avoid learning effect-related systematic errors.

## Introduction

Isometric maximal strength is defined as the capacity to generate force or torque with a voluntary isometric muscle contraction in which no joint movement takes place (Gallagher et al., 2004; Gabriel et al., 2006). Isometric maximal strength testing methods are often used in laboratory studies (Wilson, 2002) to gather more specific and/or highly precise data related to force generation capabilities.

Isometric testing is a simple and inexpensive process that is easily controlled and performed quickly. Moreover, it allows conducting strength tests at various joint angles throughout the range of motion (Brown et al., 2001; Gallagher et al., 2004; Kroemer, 1999). Isometric strength testing has been demonstrated to provide a more precise estimate of strength measure, in addition to being highly reliable and safer than isoinertial dynamic testing methods (Blazevich et al., 2006; Gallagher et al., 1998; Abernethy et al., 1995). Furthermore, isometric strength testing is more appropriate in experimental design studies than isoinertial testing because of its highly controllable features.

In isometric testing protocols, variables such as the duration of contraction, time interval over which force or torque is calculated, number of trials, rest interval between successive trials, joint angle(s), posture, type of postural control, equipment used, instructions given to participants, participants’ physical state, and environmental state can influence the variability of test results (Gallagher et al., 1998; Brown and Weir, 2001). However, no specific standardized guidelines regulating these variables are available (Brown and Weir, 2001). It should be considered that changes or differences in any of the aforementioned variables in different studies make comparisons between studies challenging.

It is stated that in isometric testing procedures, 3 trials (the best of 3 trials method [B3T]) are sufficient for participants to obtain their actual maximal strength values (Brown and Weir, 2001; Blazevich and Cannavan, 2006). However, Brown and Weir (2001) reported that the research community is uncertain concerning the number of trials that should be used in the assessment of isometric strength. Nevertheless, the B3T remains commonly used in scientific studies to assess maximal strength, and if the difference between the highest 2 trials is greater than 5%, then an additional trial is performed (McBride et al., 2007; Watanabe et al., 2009).

The squat exercise is commonly used to evaluate maximal strength of the lower limbs (Demura et al., 2010). Specifically, the isometric squat is used in laboratory studies to gather information about lower limb force generation (Balshaw et al., 2012; Verdera et al., 1999). In some isometric squat-related studies, at most 3 (rarely 4) maximal isometric squat trials were used without pre-familiarization in the assessment of maximal isometric squat strength (MISS) (McBride et al., 2007; Dumke et al., 2010; McBride et al., 2006; Nuzzo et al., 2008; Rahmani et al., 2001). However, there is no scientific evidence in the literature demonstrating that a range of 1–3 trials is sufficient to obtain MISS values that include no systematic error resulting from acute learning effects.

A pre-familiarization session is an important issue to be considered in the assessment of isometric strength measures. In human testing procedures, the intra-individual variability of the participants should be taken into account. The technique used during the test, learning effects, fatigue, motivation, verbal encouragement, visual feedback, and instructions received by the participants are some of the factors related to variability (Verdera et al., 1999; Hopkins, 2000). Scientific studies in sports medicine and exercise science are generally conducted to identify the possible effects of an intervention. It is of great importance to minimize systematic errors resulting from learning effects (training effects) prior to the start of the study. Performing sufficient numbers of testing trials is the simplest and most appropriate method of minimizing learning effects, thus avoiding systematic errors in the study results (Hopkins, 2000). This process is known as familiarization, which is a profound component in the assessment of the “actual” maximal baseline values of participants in performed tests (Wallerstein et al., 2010).

Assessing intraclass correlation coefficients (ICCs) is a common method used in reliability studies. In addition, in traditional pre-test/post-test design studies, investigators generally report ICCs of repeated measurements obtained from performance tests used in the study to indicate that their measurements are reproducible and their study results include no systematic error. However, interpretation of the reliability solely based on ICCs has various disadvantages. As the ICC is a relative reliability measure, it cannot discriminate systematic errors from random errors (Atkinson et al., 1998; Weir, 2005; Hopkins, 2000; Bland et al., 1999). In addition, the ICC is also affected by sample size and variability between the test measures of the participants (Atkinson and Nevill, 1998; Hopkins et al., 2001; Morrow et al., 1993). This issue was demonstrated clearly in a review article by Weir (2005) using a hypothetical data set. If the inter-individual variability in a study sample is large, then the ICC could be extremely high even if the repeated measurements of the sample are extremely different (low reliability in reality). If the inter-individual variability in a study sample is small, then the ICC could be small even if the repeated measurements of the sample are slightly different (high reliability in reality) (Weir, 2005). In studies by Hopkins (2000) and Hopkins et al. (2001), it was stated that analysis of reliability using the coefficient of variation (CV) and changes in repeated measurements would be more appropriate. Therefore, interpreting reliability measures based only on ICC values could be misleading as well as result in biased study results. In addition, ignoring the possible differences between repeated measurements or between measurements obtained using different measurement methods could result in drawing inaccurate conclusions from the results of a study, even if the repeated measurements are classified as highly reliable according to ICC values.

Although it has been stated that reliability statistics should be reported with their confidence limits (Hopkins, 2000), generally, confidence limits are not reported in studies in which ICCs are used as indicators of reliability, nor is any absolute reliability measure indicated. In addition, researchers fail to clarify whether the repeated measurements are significantly different.

The aims of this study were to identify 1) whether measurements obtained from a commonly used MISS assessment method (B3T) without pre-familiarization are reliable, 2) whether continuation of the B3T by performing additional trials could elicit an acute familiarization effect on the force generation potentials of participants and statistically increase the obtained MISS, 3) whether repeated measurements classified as reliable according to ICCs are sufficient for drawing accurate conclusions from isometric squat-related study results, and 4) how many trials are needed in a MISS test if no pre-familiarization is used.

## Material and Methods

### Participants

Forty males with experience in resistance training (age: 22.6 ± 3.0 years, body height: 183.4 ± 7.8 cm, body mass: 83.8 ± 11.6 kg, relative MISS: 1.77 ± 0.43, resistance training experience: 5.5 ± 3.6 years, training volume: 9.9 ± 5.9 h/week) volunteered to participate in this study. None of the participants had prior familiarization with the MISS test. They had no history of injury or health problems likely to compromise MISS.

The participants were required to refrain from intensive physical activity and the consumption of alcohol, any food or drinks containing caffeine, and any other types of stimulants for at least 24 h (Krishnan et al., 2009) prior to the testing session. In addition, they were asked to have their usual amount of nightly sleep and follow their normal diets (consumption of a light meal at least 3 h prior to testing). The participants were informed of the purpose, procedures, and experimental risks of the study. Then, each of them signed a written informed consent form, which was reviewed and approved by the Medical Ethics Committee of the Medical Faculty of the Ege University in accordance with the Declaration of Helsinki (approval number: 11-7/12). Testing sessions were conducted in the fitness center of the Ege University School of Physical Education and Sports.

### Warm-up procedure

The participants performed a standardized warm-up consisting of 2 min of walking and 6 min of running at a self-selected pace on a treadmill. Afterwards, they followed a standardized dynamic stretching protocol directed by one of the researchers (the same researcher in each testing session), as static stretching procedures have detrimental effects on isometric force production (Herda et al., 2008).

In the specific warm-up, the subjects were asked to perform 8–10 dynamic squats with an unloaded Smith machine (ESJIM ES 450 Multipress Station, ESJIM Ltd., Eskişehir, Türkiye) bar resting on their shoulders. During these squats, their self-selected squat stance positions were adjusted and marked on the floor to ensure that each participant maintained the same position throughout the testing session. After dynamic squats, isometric submaximal squat repetitions corresponding to 60, 70, 80, and 90% of the participants’ self-estimated maximal efforts were performed with 45–60 s rest periods between repetitions.

### Positioning procedure

Participants were allowed to perform isometric squat trials with a self-selected stance position to maximize their comfort during their maximal efforts.

The Smith machine bar was positioned at a height (sensitivity of 0.02 m) that permitted an approximately 90° knee joint angle (Newton et al., 2002; Alegre et al., 2006) using adjustable length chains attached to each end of the bar. Strain gauges (DESIS, CR Series Digital Crane Scales 200, Shenzhen West-Boao Science and Technology Co., Shenzhen, China) were placed in a series with each chain to record the tensile force generated during the maximal isometric squat trials. In our pilot testing, we discovered that participants achieved higher MISS in isometric squats with their hands off the bar. Therefore, they were not allowed to hold the bar during the isometric pushing phase to ensure that the recorded measurements were not affected by the weight transfer of the arms and any possible pushing or pulling forces exerted on the bar by the upper body muscles. To standardize the positions of the hands and arms, the participants were instructed to hold their hands together behind their bodies. This was a practical precaution to avoid possible systematic errors that could affect the study results. The Smith machine bar was wrapped with a mat to protect the cervical region of the subjects.

### MISS test

The MISS test started 3 min after the end of a specific warm-up. The participants were allowed to consume water ad libitum during testing. They wore a weightlifting belt during each maximal trial to support the lower back. They were instructed to exert force upward against the immovable bar as fast as possible (Rahmani et al., 2001; Alegre et al., 2006) using their maximal effort and attempt to increase this force as long as possible during each trial. Strong verbal encouragement was provided to each participant by using the same word repetitively (e.g., push, push, push, push) throughout each trial. A similar voice tone was maintained during this procedure. It was assumed that the motivation level of each participant was similar throughout the study. When a significant force decrease was detected on the strain gauges, participants were informed to stop exerting force (Markovic et al., 2004, 2007). The sum of the maximal measurements detected on each strain gauge and the mass of the Smith machine bar and chains were normalized to the body mass of the participants and defined as the relative maximal isometric squat strength (R-MISS).

The participants performed 8 isometric squat trials with maximal effort. As stated in a study by Brown and Weir (2001), 2-min rest intervals between trials should be used if a large number of isometric trials are to be performed. Therefore, trials were separated by 2-min rest intervals. However, the rest interval after the 4^th^ trial was extended to 5 min. This adjustment was performed to avoid potential cumulative fatigue, a major source of systematic error (Hopkins, 2000), resulting from the first 4 maximal efforts, and ensure that participants were fully recovered. Thus, participants were able to exert maximal efforts across the subsequent trials. If the highest measure was obtained in the 7^th^ or 8^th^ trial, participants were asked to perform 2 additional trials under the assumption that they could perform better and obtain a higher force measurement (which would be their real MISS) in these additional trials. The aim of this process was to ensure that the subjects’ force generation potential reached a plateau. The test procedure was continued until the measurements obtained in the last 2 trials of the participants were lower than the highest measurement obtained in the previous trials. Participants who performed more than 8 trials rested 5 min after the completion of the 8^th^ trial based on the same premise of the 5-min rest interval provided after the 4^th^ trial. MISS obtained in the 9^th^ or later trials was regarded as if it were obtained in the 8^th^ trial for the statistical analyses. In this study, the MISS values obtained in the first 3, 4, 5, 6, 7, and 8 (or more) trials were recorded, and each measurement was regarded as that obtained from different MISS assessment methods based on the numbers of trials, which were named as the B3T, best of 4 trials method (B4T), best of 5 trials method (B5T), best of 6 trials method (B6T), best of 7 trials method (B7T), and best of 8 trials method (B8T), respectively. In this study, B3T and B8T were regarded as the criteria against which all other methods were compared.

### Statistical analyses

The R-MISS data of this study were analyzed using IBM^®^ SPSS^®^ Statistics for Windows version 20 software (Armonk, NY: IBM Corp., 2011). The Shapiro-Wilk test was performed, and histograms with a normal curve were checked to assess the normality of related data. The non-parametric Wilcoxon signed-rank test was used to assess whether the mean ranks of paired methods differed because the normality assumption was violated. An α level of p ≤ 0.003 was considered statistically significant after the Bonferroni correction for all possible pairwise comparisons between methods (p = 0.05/15). The effect size (r) for each comparison was also presented.

The ICC and 95% confidence intervals (95% CIs), as well as the CV values, between paired methods were calculated on the basis of the natural logarithm-transformed R-MISS data. The ICCs were computed using 2-factor mixed-effects single-measure reliability (absolute agreement). The Bland-Altman method was used as an absolute reliability statistic to assess agreement and the disparity between paired methods, as it separated systematic and random errors (Bland and Altman, 1999). The acceptable agreement limit was set at 5%.

Replications of this study on several different samples with different sizes are needed to identify the minimum number of trials necessary for assessing the actual MISS of participants within a single testing session without pre-familiarization. Therefore, simple simulation studies were performed starting with the assumption that the sample of this study was a good representative of the resistance-trained athlete population. The data of each participant were embedded into a separate row in a Microsoft^®^ Office 2007 Excel worksheet. Four hypothetical groups with different sample sizes (n = 8, n = 12, n = 24, and n = 30, respectively) were randomly constituted among the 40 participants of this study via an Excel macro. This process was repeated 500 times for each sample size. The distribution of these hypothetical groups was analyzed using frequency analysis (90^th^ percentiles) according to the total number of trials needed for at least 90% of participants in these groups (10% was assumed to be an acceptable error level) to obtain their actual MISS. This distribution was used to calculate the probabilities that “at least 90% of participants reach their actual MISS” in studies with specified sample sizes and with different numbers of isometric squat trials.

## Results

In total, 15 of the 40 participants performed more than 8 trials in their MISS assessments according to the test protocol. It was found that participants obtained significantly higher R-MISS values in B8T (Mdn = 1.69) than in B3T (Mdn = 1.66) (z = 4.62, p < 0.001, r = 0.73), illustrating that 28 participants (70%) increased their R-MISS in the former. In addition, all pairwise comparisons between criterion methods (B3T and B8T) and other methods displayed significant differences (p < 0.001) excluding B7T-B8T (p = 0.012), indicating that higher R-MISS values were obtained in methods including more trials than in those including fewer trials (Table 1).

**Figure 1:**
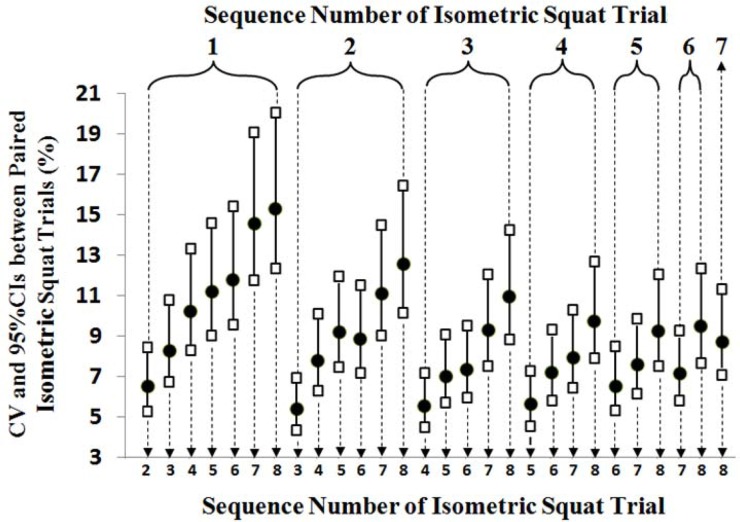
Coefficients of variation (CV) and 95% confidence intervals (CIs) among the log-transformed data of 8 relative maximal isometric squat strength measurements obtained in the study Circles and squares represent CVs and 95% CIs, respectively. The numbers above the brackets at the top of the graphic indicate the sequence number of the isometric squat trial paired with other trials (represented by dotted arrows) for the statistical analyses

**Figure 2:**
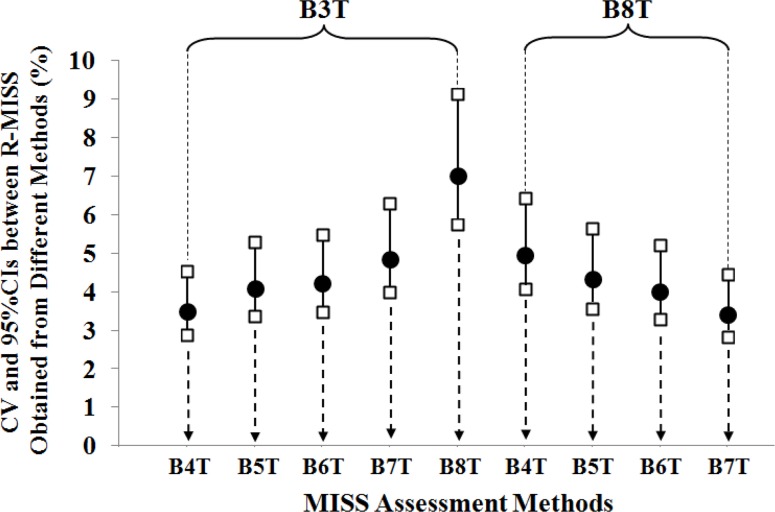
Coefficients of variation (CV) and 95% confidence intervals (CIs) among the log-transformed data of relative maximal isometric squat strength (R-MISS) measurements obtained using different maximal isometric squat strength (MISS) assessment methods Circles and squares represent CVs and 95% CIs, respectively. The Best of 3 Trials Method (B3T) and Best of 8 Trials Method (B8T) were paired with the other methods (represented by dotted arrows) for the statistical analyses. B4T = The Best of 4 Trials Method; B5T = The Best of 5 Trials Method; B6T = The Best of 6 Trials Method; B7T = The Best of 7 Trials Method

High ICCs [95% CIs] (range of 0.93 [0.86–0.96] to 0.98 [0.97–0.99]) and low CVs [95% CIs] (range of 3.4 [2.9–4.5%] to 7.0% [5.7–9.1%]) were found between the paired methods (Table 1). In addition, high ICCs [95% CIs] and low CVs [95% CIs] were observed between successive isometric squat trials (ICC range: 0.91 [0.84–0.95] to 0.96 [0.93–0.98]; CV range: 5.4 [4.4–7.0%] to 8.8% [7.1–11.4%]) (Table 1 and Figures 1–2). In addition, no statistically significant difference was found in R-MISS between successive trials (p = 1.00), excluding the 1st and 2nd trials (p = 0.003).

The Bland-Altman method revealed that no acceptable agreement was found between any of the compared methods. The relative limit of agreement (LoA) values ranged from 12.4 to 24.5% and from 9.3 to 25.5% when methods were compared with B3T and B8T, respectively (Table 1).

Only 30% of the participants obtained their MISS in B3T. More than 50% of them required 6 or more trials to obtain their actual MISS. When the highest 2 measurements of the participants were considered, 45, 30, and 27.5% of the subjects obtained their highest 2 measurements in the 8th, 7th, and 5th trials, respectively. Fifty percent of the participants obtained their lowest measures in the 1st trial. In addition, when the lowest 2 measurements of participants were considered, 60, 27.5, and 27.5% of the participants obtained their lowest 2 measurements in the 1st, 2nd, and 7th trials, respectively (Table 2).

When the data set of this study was evaluated in the context of commonly used maximal isometric strength assessment procedures based on B3T and a 5% critical limit (if the difference between the highest 2 measurements exceeds 5%, an additional trial is to be performed), the results of MISS testing in this study would be interpreted as follows. The difference between the highest 2 measurements of 29 participants in B3T would be within 5%. However, only 10 of these 29 participants would reach their actual MISS in the B3T. Nineteen participants would not require an additional trial; as a result, their MISS would be underestimated. The remaining 11 participants would perform an additional trial (B4T). Only 4 of them would meet the criterion of the 5% critical limit in the 4th trial; however, none of them would reach their actual MISS. The remaining 7 participants would attempt a 5th trial (B5T), and 4 of them would meet the criterion of the 5% critical limit. However, as observed in the 4th trial, none of them would reach their actual MISS. Only the remaining 3 participants would reach their actual MISS and meet the 5% critical limit. In total, only 13 of 40 participants (32.5%) would reach their actual MISS, whereas the MISS of 27 participants (67.5%) would be underestimated according to commonly used maximal isometric strength assessment procedures, which could be regarded as a profound bias for a scientific study.

**Figure 3:**
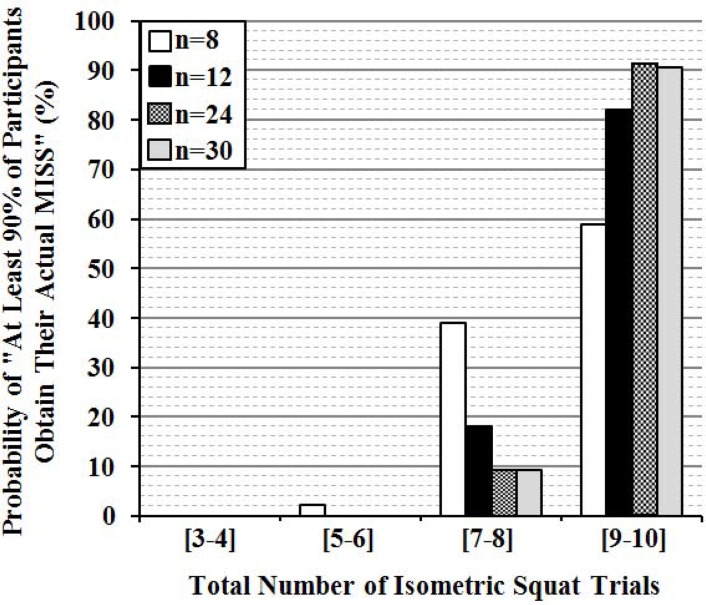
Probabilities of obtaining actual maximal isometric squat strength (MISS) measurements depending on the numbers of isometric squat trials performed in studies conducted with different sample sizes (n = 8, n = 12, n = 24, n = 30) without pre-familiarization

The results of the simulation studies revealed that in case of a study design including 30 participants, the probability that “at least 90% of these 30 participants would obtain their actual MISS” is 90.6% if 9–10 trials are performed. These probability values for study designs with sample sizes of 24, 12, and 8 were 90.8, 82.0, and 58.8%, respectively. However, if the number of trials is reduced to 7 or 8 trials, these probability values deeply decline to 9.4, 9.2, 18.0, and 39.0% for sample sizes of 30, 24, 12, and 8, respectively (Figure 3).

## Discussion

To our knowledge, this is the first study to question whether the results of isometric squat-related studies conducted within a single testing session without pre-familiarization using a limited number of trials might have included systematic errors caused by acute learning effects. The major findings of this study were as follows: 1) MISS obtained from a commonly used method (B3T) without pre-familiarization was reliable in terms of ICC and CV values; 2) the continuation of B3T by performing additional trials appeared to elicit acute learning effects on the force generation potential of the participants; 3) although MISS obtained from B3T and other methods could be classified as reliable when evaluated only on the basis of ICC values, 3 trials were not sufficient to draw accurate conclusions from isometric squat-related study results because the MISS values obtained from methods including more than 3 trials were statistically higher than those obtained from B3T; and 4) it would be better to use at least 9–10 trials with a relatively large sample size (i.e., ≥ 24) to obtain unbiased results from isometric squat-related studies without pre-familiarization.

It is of great importance that investigators report reliability levels between repeated trials used in the assessment of the baseline performance measurements of participants, especially in pre-test/post-test design and independent group design studies. In this manner, investigators can ensure that repeated measurements, and thus their study results, are not affected by systematic errors. It has been stated that CVs and differences between repeated measurements are the most important indicators of reliability (Hopkins, 2000). However, investigators generally report “only” ICC values with no confidence interval as the measure of reliability in their studies (Hopkins, 2000). In addition, in several isometric squat-related studies, limited numbers of trials were used without pre-familiarization, and ICC values were not reported as a reliability statistic (Alegre et al., 2006; Cormie et al., 2006; Dumke et al., 2010; McBride et al., 2006; Newton et al., 2002).

In our study, MISS obtained using different methods including different numbers of trials was found to be highly reliable, as indicated by high ICC and low CV values. However, the conclusion that “there is no need to use more than 3 trials as B3T is as reliable as other methods including more trials” could be misleading because MISS obtained from B3T was found to be statistically less accurate than those obtained from other methods.

As expected, LoA between paired methods revealed that the worst agreement was noted between B8T and B3T, whereas the best agreement was measured between B8T and B7T. However, this best agreement limit could not be regarded as an acceptable agreement limit, as the value of this limit (9.3%) was larger than the predetermined 5% critical limit. However, Hopkins (2000) stated that LoA in the Bland-Altman method constitutes very large thresholds in practice and suggested that the use of half of these values would be more appropriate. If LoA in this study was evaluated according to Hopkins (2000), it would have been found that large numbers of trials (7 trials) were still needed to reach the predetermined LoA because only the half of the LoA values between B8T and B7T (9.3/2 = 4.65%) would have been less than 5%. This result would be consistent with the result of pairwise comparison between B8T and B7T indicating no statistically significant difference (p = 0.012). On the contrary, this stabilization appears to occur due to the low significant α level (p ≤ 0.003) set after the Bonferroni correction for all possible pairwise comparisons (0.05/15). This statistically insignificant difference, however, could be interpreted as scientifically significant because 8 participants (20% of the study sample) increased their MISS after 7 trials and the effect size of this increase (r = 0.40) was moderate to large.

The repeated finding of similar graphical patterns related to variability among MISS values obtained from each trial (Figure 1) could also be interpreted as an indicator of acute learning effects in our study. It is obvious that variability decreases between measurements as the difference between the sequence numbers of compared isometric squat trials decreases. Similar patterns also existed between compared MISS assessment methods (Figure 2).

When descriptive statistics were evaluated, it was found that majority of the highest 2 measurements of the participants were obtained in the last 4 trials, indicating a possible learning effect over the course of the MISS test. By contrast, the majority of the lowest 2 measurements of participants were obtained in the first 4 trials, especially in the 1st trial, likely due to the unfamiliarity of the participants with the MISS test. Although a relatively high percentage of participants also obtained one of their lowest 2 measurements in the 7th trial, this may have been caused by a possible loss of interest or motivation in some participants (Hopkins, 2000), as large numbers of participants also obtained one of their highest 2 measurements in the 7th trial. This finding was also supported by the CVs obtained in the study. Although the lowest CV was expected to be found between the isometric squat trials performed at the final stage of the MISS test (7th and 8th trials) due to minimization of the learning effect, this was not the case, possibly due to high intra-individual performance changes in the 7th trial, as demonstrated by the descriptive statistics (Table 2).

In his book, Kline (2004) wrote that “(…) In any science, though, it is replication that is the ultimate arbiter: No matter how intriguing a result from a single study, it must be replicated before it can be taken seriously. Replication also is the ultimate way to deal with the problem of sampling error (...)”. Therefore, we performed simple simulation studies and determined some probability values related to the required number of isometric squat trials to obtain an unbiased MISS. Starting with the assumption that our sample was a good representative of the resistance-trained athlete population, it was concluded at least 9–10 trials with a relatively large sample size (i.e., ≥ 24) should be used in a MISS test to ensure that the values in studies without pre-familiarization were not affected by systematic errors (provided that an underestimation of MISS among 10% of the study sample is an acceptable error level). Depending on this conclusion, it could be argued that investigators who assessed MISS using limited numbers of trials without pre-familiarization in their study might have obtained results that included systematic errors. Therefore, the results of these studies should be evaluated with caution.

Whitley and Elliot (1968) and Green et al. (2013) found that the learning effect during isometric contractions was completed within at least 6 and 10 trials, respectively. These results which emphasize the use of relatively large numbers of trials to obtain stabilized and reliable isometric maximal strength measurements during a single testing session, are in line with our findings.

It is rational to state that each trial in the MISS test in this study appeared to have served as a means of acute familiarization for further trials. Participants might have learned how to activate their muscles more efficiently after each trial. It has been stated that this learning effect is observed in both untrained and trained individuals (Dias et al., 2005). The learning effect was stated to be substantial for static contractions that include complex muscular activation strategies (Whitley and Elliot, 1968). From a physiological point of view, it is unlikely that improvement in the MISS of participants in the present study resulted from a post-activation potentiation (PAP) effect because there was no sufficient evidence supporting such an effect in maximal force-related performances. Additionally, it was stated that PAP had little effect on high-force/low-velocity movements such as isometrics (Tillin et al., 2009). Improved intraand inter-muscular coordination (Calder and Gabriel, 2007; Gabriel et al., 2006; Whitley and Elliot, 1968) could be the main sources of learning effects that result in increased force generation capabilities across testing trials and sessions. Although no physiological measurement was performed in this study, the mechanism of learning effects was attributed to neural factors such as increased firing rates of motor neurons, increased activity of agonist muscles, decreased co-contraction of antagonist muscles, adaptation in motor cortical processes, and familiarization with the biomechanical pattern of the movement (Calder and Gabriel, 2007; Gabriel et al., 2006; Green et al., 2013; Kamen and Knight, 2004).

Rapid adaptation of the central nervous system, particularly for complex movements with which individuals are not familiar, is difficult to achieve within a limited number of testing trials. Therefore, extensive familiarization is necessary for the accomplishment of a full learning process, especially when the performance tasks include activation of large muscle groups (e.g., quadriceps, pectoralis major) (Dias et al., 2005). This is probably the most rational explanation of the need for a relatively large number of trials among the study participants to obtain their actual MISS measurements.

### Methodological limitations

The most important limitation of this study was the use of strain gauges rather than a force platform in the assessment of MISS. It could be questioned whether systematic errors based on the use of a different measurement tool were present in the study. Although it is difficult to discriminate measurement tool-source errors from biological errors, it is unlikely that our results were affected by systematic errors, largely because the reliability statistics between measurements obtained from successive isometric squat trials suggested high reliability. In addition, no statistically significant difference was found between successive trials excluding the first 2 trials (p = 0.003), which might have been due to the immediate learning effect after the first trial, as 50% of the participants obtained their lowest measure in this trial.

The testing posture used in the traditional isometric squat test and that used in our study (no handhold) were different. This issue could be considered a factor that makes comparisons between our results and those of other studies difficult. However, the testing posture used in our study likely avoided any possible forces exerted on the bar by the use of any upper body muscle(s). Thus, this squat posture served as a practical method to avoid systematic errors that might have been included in other studies. Because the isometric squat test is used to gather information about lower body force generation (Balshaw and Hunter, 2012; Verdera et al., 1999), the testing posture should be adjusted appropriately to ensure that no muscle other than those of the lower body is involved in the force generation phase.

In conclusion, the common use of B3T in MISS tests without pre-familiarization appears to have no solid scientific foundation, as relatively large numbers of trials (≥ 8 trials) were needed for participants to obtain their actual MISS in this study. Accordingly, using large numbers of trials with long rest intervals, which could constitute an effective means of avoiding systematic errors (fatigue and learning), especially in testing sessions without pre-familiarization, appears to be a practical implication for isometric tests. This implication could prevent investigators from underestimating the baseline values of participants and overestimating the intervention effects in studies without pre-familiarization. Limiting trial numbers after obtaining high reliability levels based on ICC values “only” could be misleading; by contrast, assessment of possible differences between repeated measurements should be given priority. It is of great importance to note that the conclusions drawn from the results of studies in which MISS assessments were performed using a limited number of trials without pre-familiarization should be interpreted with caution, as these results might have been affected by systematic errors, possibly due to underestimated baseline measurements. Questioning the assumptions of scientific studies and testing these assumptions experimentally are the most important responsibilities of investigators (Kunz et al., 1998). Therefore, researchers should ensure that the testable assumptions of their planned studies are valid. Unless the validity of these assumptions is proved, doubts may arise concerning the reliability of obtained study results.

Conducting similar studies on different muscle groups (small and large) using different exercises (single-joint and multi-joint) will provide valuable knowledge for the standardization of isometric tests commonly used in the fields of sports medicine and exercise science. This issue is crucial for both investigators and coaches who use isometric tests in the assessment of maximal strength levels of their athletes. The use of isometric tests with appropriate procedures allows coaches to assess the strength levels of their athletes accurately and compare strength measurements with previously obtained values to detect possible strength improvements without systematic errors.

## Figures and Tables

**Table 1:** Statistical results indicating relative (ICC) and absolute (CV, LoA) reliability and the significance of the mean rank difference between the R-MISS values of paired methods

Paired Methods	Significance of Mean Rank Δ			Reliability Statistics			

	Change	ES	p**^†^**	ICC [95% CIs]**^‡^**	CV [95% CI] (%)	LoA [95% CI] (%)	
	
B4T-B3T	19 (+), 21 (=)	0.60	< 0.001^*^	0.98 [0.97–0.99]	3.5 [2.9–4.5]	12.4	[9.8–15.1]
B5T-B3T	22 (+), 18 (=)	0.65	< 0.001^*^	0.95 [0.97–0.99]	4.1 [3.3–5.3]	16.0	[12.8–19.3]
B6T-B3T	27 (+), 13 (=)	0.72	< 0.001^*^	0.95 [0.97–0.99]	4.2 [3.5–5.5]	17.2	[13.8–20.5]
B7T-B3T	28 (+), 12 (=)	0.73	< 0.001^*^	0.93 [0.96–0.98]	4.9 [4.0–6.3]	20.0	[16.1–23.8]
B8T-B3T	28 (+), 12 (=)	0.73	< 0.001^*^	0.93 [0.86–0.96]	7.0 [5.7–9.1]	24.5	[19.8–29.3]
B8T-B4T	23 (+), 17 (=)	0.66	< 0.001^*^	0.96 [0.93–0.98]	5.0 [4.1–6.4]	17.3	[13.8–20.8]
B8T-B5T	21 (+), 19 (=)	0.63	< 0.001^*^	0.97 [0.94–0.98]	4.4 [3.6–5.6]	13.6	[10.7–16.4]
B8T-B6T	15 (+), 25 (=)	0.54	< 0.001^*^	0.97 [0.95–0.99]	4.0 [3.3–5.2]	11.9	[9.3–14.5]
B8T-B7T	08 (+), 32 (=)	0.40	<0.012	0.98 [0.97–0.99]	3.4 [2.8–4.4]	9.3	[7.1–11.4]

* p < 0.001;

† p ≤ 0.003 is the significant α level in the context of the Bonferroni correction for all possible pairwise comparisons between methods (p = 0.05/15).

‡ All ICC values are significant at the level of p < 0.001; Δ = Difference; (+) = Number of participants who increased their R-MISS measurements; (=) = Number of participants who had no change in their R-MISS measurement; B3T = The Best of 3 Trials Method; B4T = The Best of 4 Trials Method; B5T = The Best of 5 Trials Method; B6T = The Best of 6 Trials Method; B7T = The Best of 7 Trials Method; B8T = The Best of 8 Trials Method Increment; CI = Confidence Interval; CV = Coefficient of Variation calculated on the basis of log-transformed data; ES = Effect Size; ICC = Intraclass Correlation Coefficient calculated on the basis of log-transformed data; LoA = Limit of Agreement; R-MISS = Relative Maximal Isometric Squat Strength

**Table 2:** Distribution of the number of participants according to the trial number and ranking of the obtained strength measurements

		First Series				Second Series			

		1^st^ Trial	2^nd^ Trial	3^rd^ Trial	4^th^ Trial	5^th^ Trial	6^th^ Trial	7^th^ Trial	^*^8^th^ Trial

		n (%)				n (%)			
Ranking of Obtained Strength Measures	Highest	2 (5.0)	5 (12.5)	5 (12.5)	5 (12.5)	2 (5.0)	6 (15.0)	7 (17.5)	8 (20.0)
	2^nd^ Highest	5 (12.5)	3 (7.5)	2 (5.0)	3 (7.5)	9 (22.5)	3 (7.5)	5 (12.5)	10 (25.0)
	3^rd^ Highest	2 (5.0)	5 (12.5)	6 (15.0)	10 (25.0)	2 (5.0)	5 (12.5)	6 (15.0)	4 (10.0)
	4^th^ Highest	0 (0.0)	4 (10.0)	4 (10.0)	8 (20.0)	10 (25.0)	9 (22.5)	2 (5.0)	3 (7.5)
	4^th^ Lowest	3 (7.5)	4 (10.0)	6 (15.0)	5 (12.5)	4 (10.0)	9 (22.5)	5 (12.5)	4 (10.0)
	3^rd^ Lowest	4 (10.0)	8 (20.0)	8 (20.0)	4 (10.0)	6 (15.0)	2 (5.0)	4 (10.0)	4 (10.0)
	2^nd^ Lowest	4 (10.0)	9 (22.5)	6 (15.0)	4 (10.0)	4 (10.0)	4 (10.0)	6 (15.0)	3 (7.5)
	Lowest	20 (50.0)	2 (5.0)	3 (7.5)	1 (2.5)	3 (7.5)	2 (5.0)	5 (12.5)	4 (10.0)

* Maximal isometric squat strength measurements obtained in the 9^th^ or later trials were regarded as if they were obtained in the 8^th^ trial.
